# ‘The Norm Is to Not Openly Collaborate’: Using the Lens of Co‐Production to Evaluate the Development of a COVID‐19 ICU Triage Policy

**DOI:** 10.1111/hex.14159

**Published:** 2024-07-27

**Authors:** Brett Scholz, Flick Grey, Joyce Graham, Imogen Mitchell, Lucy Kirk, Terri Warner

**Affiliations:** ^1^ School of Medicine and Psychology The Australian National University Ngunnawal and Ngambri Country Canberra Australian Capital Territory Australia; ^2^ Affiliated along my own idiosyncratic lines Wurundjeri Country Melbourne Victoria Australia; ^3^ Aboriginal and Torres Strait Islander Liaison Service Canberra Health Services Ngunnawal and Ngambri Country Canberra Australian Capital Territory Australia; ^4^ Research and Academic Partnerships Canberra Health Services Ngunnawal and Ngambri Country Canberra Australian Capital Territory Australia

**Keywords:** co‐production, consumer leadership, COVID‐19, lived experience leadership, triage

## Abstract

**Introduction:**

In 2020, surging cases of COVID‐19 meant that health services had to plan for crisis‐level triage. In the Australian Capital Territory, the Clinical Health Emergency Coordination Centre sought to develop a triage policy in collaboration with a range of consumer, carer and community groups. This study aims to map the collaborative development of the COVID‐19 ICU triage policy onto the principles of co‐production.

**Methods:**

Interviews were conducted with facilitators, members of advocacy or consumer groups and clinicians who were involved in the development of the triage policy. Interviews were thematically analysed using both theory‐ and data‐driven approaches to, respectively, draw on the theoretical framework of co‐production, and to explore participants' perspectives relevant to but beyond the scope of this theoretical framework.

**Results:**

The findings suggest that at each stage of the initiative, there were ways in which the principles of co‐production were met, and ways in which they were not met. One of the fundamental concerns that arose was about whether trying to solve a problem based on resources was compatible with a solution based on human rights.

**Conclusion:**

Literature about co‐production has been critiqued for being limited to aspirational concerns, or implying co‐production is easily achievable. The current study contributes to existing research through the application of the theoretical framework of co‐production and exploring ways its aims were met and not met within a system‐level collaboration developing a high‐stakes health policy.

**Patient or Public Contribution:**

This study has been conducted and written by researchers working from lived experience perspectives, and other researchers working from traditionally mainstream health disciplines, including psychology and medicine. Further, the study is about patient and public involvement in the development of a health policy. Thus it both embodies and is about non‐tokenistic collaboration between people with lived experience and other health professionals.

## 1 Introduction

The COVID‐19 pandemic led to large numbers of people needing critical care services in healthcare systems around the world—and in several jurisdictions, these needs exceeded capacity and contingency surge strategies [1]. With surging cases of COVID‐19 across jurisdictions, health services began planning for crisis‐level triage. Both internationally and in Australia, there had been significant concerns that health directives and medical decision‐making protocols developed amidst the pandemic were not based on equity [2, 3].

Advocates suggest that the people with crucially needed knowledge are those with direct (or indirect) experience of the matter being investigated— including patients and carers with an experience of a particular condition, or members of the public at risk of a particular condition [4]. In the context of this pandemic, while the whole population was at risk of contracting COVID‐19, not all people had the same risk—and in particular some groups were at a greater risk of higher morbidity and mortality, and systemic and social discrimination [5, 6].

### 1.1 Study Context

The numbers of COVID‐19 patients in Australia in 2020 were generally lower than in many other countries (although with differences across and within Australian jurisdictions as outlined by Storen and Corrigan [7]). In the Australian Capital Territory (ACT), before May 2020, an initial decision‐making framework for the allocation of acute care was rapidly developed internally by the local health service. This was produced quickly with external stakeholders (including consumer organisations and groups) having just a few days to respond to consultation about the framework.

By May 2020, the Clinical Health Emergency Coordination Centre in the ACT realised that there was more time to start a new and develop a triage system in collaboration with consumers, carers and community members [8]. By invitation of the Clinical Health Emergency Coordination Centre, the Health Care Consumers Association of the ACT and local university academics collaborated to formulate a strategy to ensure that community members had control over the development of a triage process for the allocation of intensive care unit (ICU) resources [9]. It is this collaboratively designed triage system that is the focus of the current study.

### 1.2 Theoretical Framework

The theoretical framework for the current study is based on the philosophical approach used in the development of the triage process: co‐production with consumers of health services [8]. Addressing criticisms about other forms of consumer participation, co‐production purports to be a different type of partnership based on dialogue and learning [10]. An ongoing and growing criticism of initiatives that claim to be based on co‐production—or other collaborative methods—is that such terms are used vaguely or tokenistically (e.g., claiming to have engaged in ‘partnership’ when in fact something more like consultation has been done [11]). To consider ways in which the aims of co‐production had been met in producing the principles and process for triage, we drew on the three core principles of co‐production [12]:

a. that consumers should be partners from the beginning of and throughout the process;
b. that power differentials should be acknowledged, explored and addressed; and
c. that consumer leadership and capacity should be developed.

Much of the academic literature to date about co‐production has been aspirational [13], and thus the current study seeks to evaluate an attempted application of these principles of co‐production during the development of an ICU triage process across a health service for the COVID‐19 pandemic.

### 1.3 Aim and Objectives

The aim of this study was to map the collaborative approach taken to the development of the triage process for the allocation of intensive care resources during the COVID‐19 pandemic in the ACT on to the principles of co‐production. To achieve this aim, the specific objectives were to:

a. collect stakeholders' accounts of the collaborative approach;
b. compare these accounts to the principles and processes of co‐production;
c. critique the collaborative approach in relation to ways in which it met and did not meet the ideals of co‐production; and
d. draw upon learnings from this study to produce recommendations for others considering how to apply the principles of co‐production.

## 2 Materials and Methods

### 2.1 Design

This study adopted a qualitative exploratory design [14] allowing us to inductively and deductively examine the ways in which the collaborative development of the ICU triage process for the allocation of intensive care resources during the COVID‐19 pandemic in the ACT could be mapped onto the principles of co‐production.

### 2.2 Data Collection

Participants were recruited based on their involvement in the triage development process, including as facilitators (*n* = 3), specific advocacy groups or consumers (*n* = 9) and clinicians (*n* = 2). Participants were interviewed using open‐ended, semi‐structured interviews that provided them with a lot of opportunities to direct conversations important to their experiences of the collaborative process. Ethics approval was granted by the ANU Human Research Ethics Committee (#2020/272), and participants were informed that they could withdraw at any stage during the interview or decline to answer any specific question.

### 2.3 Data Analysis

Analysis was guided by the principles and process of thematic analysis [15]. This involved iterative reading and coding of data from both theory‐ (drawing on the theoretical framework of co‐production) and data‐driven perspectives (to be open to participants' experiences that were relevant but beyond the scope of current theory). Codes related to similar aspects of the data were grouped together and through a further iterative process, themes were developed and examined for relationships with the theoretical framework.

## 3 Results

Our findings suggest there were ways in which the principles of co‐production were met and ways in which they were not met in the collaborative development of the triage process for intensive care during the COVID‐19 pandemic in the ACT. Even outside the context of the pandemic working towards co‐production is complex, it is important to consider how stakeholders perceive meaningful and productive collaboration. Table 1 maps participants' accounts of the collaboration to the principles of co‐production across all phases (i.e., co‐planning, co‐design, co‐delivery and co‐evaluation). While most stakeholder groups reported that they considered the project a good example of co‐production (e.g., a representative from one organisation noted it was ‘the first time we've had an opportunity to shape one of [the local health organisation's] initiatives’), some did not (e.g., one participant said: ‘co‐production … is where … you start with a blank slate a problem to solve … and that wasn't the case here’). In each of the sections below, we discuss how the principles of co‐production were considered to have been met or not.

**Table 1:** Mapping principles of co‐production to each stage of the project.

How each stage was operationalised	Ways in which co‐production principles were met	Ways in which co‐production principles were not met
Co‐planning: Planning what problem needed a solution, which groups and individuals should be involved and how decision‐making should occur.	The engagement plan was collaboratively developed over time between academic and consumer partners (from the peak body for healthcare consumers in the territory). Discussion about governance (such as what to do about dissensus, and how to create modalities for engagement) was done collaboratively between academic and consumer peak body partners. It was decided that the individual consumer, carer and community organisations would meet collaborators' needs, both tangible (e.g., wifi support) and intangible (e.g., pastoral check‐ins). However, the facilitators would also be flexible when these needs could not be met, for instance, making alternative arrangements if wifi was unreliable or they felt safer engaging in other ways.	The engagement plan—although developed between academic and consumer peak body partners—was not collaboratively developed with all stakeholder groups. The timeframe was largely directed by the health service (although this did become more flexible because of the reduction in COVID‐19 risk throughout the project).
Co‐design: Defining and refining the problems and solutions.	Multiple ways of engaging allowed for a wide range of stakeholders to discuss and develop solutions together. The underpinning principle of the triage process was transformed through collaboration. Particular groups were more challenging to contact (in terms of finding the person/s to contact or be engaged)—particularly as the health advocacy and social service sectors were overwhelmed with COVID work at the same time. o In particular, one of the most discriminated groups in this space—i.e., older adults—was underrepresented throughout the process.	The ‘problem’ to solve emerged from the health service's concern about dealing with limited intensive care resources. Many from the disability advocacy sector suggested the question should not be about limited capacity, but that the question itself should emerge from a human rights perspective.
Co‐evaluation: Synthesising the proposed solutions into two documents (i.e., triage principles and triage processes) and examining whether the product met expectations.	Providing iterative copies of the project to collaborators progressed the changes started in the previous phase. Providing multiple modalities for people to engage (directly editing, commenting and discussions) with the emerging output allowed for progressive development.	The core principle underpinning the collaboratively developed output (i.e., non‐discrimination) was embedded but even right up to the penultimate draft, a discriminatory clause remained. Collaborators seemed reluctant to make direct edits to the documents provided. Some groups that had been invited to take part in earlier phases but who were only able to join at this phase were more disconnected from the project.
Co‐delivery: Submission of the triage principles and processes for endorsement by the Clinical Health Emergency Coordination Centre and implementation within the health service.	The steering committee formed to escalate the project to the Clinical Health Emergency Coordination Centre, although necessarily smaller than the broader collaborative process, included some key representation (specifically, a person with a disability, a healthcare consumer, a human rights commissioner, a bioethicist and a clinician from each of the two hospitals in the region). Having gone through this process allowed the steering committee to demonstrate the value and community buy‐in to the final product and to strengthen its advocacy of the project. Once implemented into policy, updates and the final triage details were sent by the Clinical Health Emergency Coordination Centre to all individuals and organisations.	People engaged in earlier stages were concerned about the lack of representation at this stage. There were three stakeholder groups of particular concern or particularly concerned at this phase: o One of the groups providing care to Aboriginal and Torres Strait Islander people was concerned about ongoing racism experienced across the health system and the implications of racism in a pandemic. o There was still no contact from the peak body for older people—although older consumers had still collaborated through other peaks or channels. o A core group of disability advocacy organisations still had reservations about several elements of the final product.

### 3.1 Principle 1: Partners From the Outset

The first principle of co‐production is about ensuring that those in positions of less institutional power (in this case consumers) are involved in setting priorities, agendas and making decisions from the beginning [12]. Participants spoke about *three* aspects of being partners from the outset: planning, communication and continual collaboration.

#### 3.1.1 Initial Collaborative Planning

The planning phase involved the iterative development of a collaborative strategy between members of the peak health consumer organisation in the ACT and members of the facilitation team from a local university—reporting to the Clinical Health Emergency Coordination Centre of the Territory. Together with the peak health consumer organisation, relevant consumer, carer and community groups were identified for collaboration. Key questions in this phase related to how to deal with limitations on time and modalities of engagement due to COVID‐19; what consumers and consumer groups needed to partner on the project, as well as process considerations such as how to deal with dissensus between partners. This co‐planning phase led to several mechanisms that made space for increased collaboration such as multiple modalities for engagement (including videoconference forum sessions, one‐on‐one videoconference meetings and written submissions [8]). It was also at this phase that the questions to be answered through the project were clarified—and these related to better understanding how local consumer, carer and community groups thought triage should be managed in the case of a severe COVID‐19 outbreak in the region.

One of the ways in which planning of the collaboration was based on partnership from the outset was that consumer, carer and community partners were invited through their relevant peak bodies or organisation. The facilitators initially emailed most organisations asking them to consider in which ways they would like their members to be engaged. Some organisations were advocacy‐focused and thus did not have members or service users per se, and instead, their own employees took part; for other organisations, there were key service users they knew would be interested and keen to be engaged; and another set of organisations sought feedback from consumers and members and brought a synthesis of this to the collaborative discussions.

A concern arising throughout the project with some collaborators from the disability advocacy sector was about a mismatch between their approach, and the way that the health system partners were approaching the fundamental question of the collaboration exercise (i.e., in relation to how best to allocate critical care resources). While the health system partners were concerned about knowing what to do in the event of a lack of ICU capacity, the disability advocacy sector placed greater emphasis on ensuring equity and access to all. One participant noted that this fundamental difference was still present in the ‘second to last draft’, and that it was not until the final draft that this difference was resolved (and this is discussed further in Extract 5 in a subsequent section). Thus, even when collaboration has occurred from the start and throughout a project or initiative, partners may have very different views about fundamental considerations.

#### 3.1.2 Maintaining Continual Collaboration

One of the ways in which the emphasis on partnerships from the outset (and through the life of the project) was applied was through ongoing collaboration as an iterative process. This involved the facilitation team drafting a set of principles based on initial engagement with the groups of consumer, carer and community organisations' members. Iterations of the triage documents were then sent back to partners to ensure the product was closer to meeting their needs and expectations. These documents included reports on the discussions with stakeholders, the synthesis of principles derived from these discussions and the technical triage process drafts. From the facilitators' perspective, having consumer, carer and community partners edit the documents directly would have been ideal, as in Extract 1.

Extract 1.

Maybe because the norm is to not openly collaborate, stakeholders assume that what is being sought is feedback rather than action … Even when we specifically asked for people to make changes directly, most only provided feedback or comments. I saw everyone as an equal owner of the document, but I think in our sector, consumers are so conditioned to not being able to take ownership … I then have to try and interpret what they mean by the comment, interpret how they would like that … feedback to … inform the document and then make that change. If they felt empowered to make the change in the first place, it gets rid of a lot of the interpretation and possibly misinterpretation.

Much of the iterative development of the triage process involved feedback rather than direct edits, as discussed in Extract 1. For some, this may have been a matter of time (it was in the middle of dealing with the beginning of the pandemic), but the participant in Extract 1 suggests there may be norms inhibiting making direct changes to outputs. Thus, it may be important to be very clear with collaborators that they can (and should) feel they can take control, which in this case would be directly editing the document. This is an issue that might require significant thought in the planning stages of projects because writing or producing outcomes collaboratively is challenging even when there are no power differentials. Further, at the time of this collaboration, there were COVID‐19 lockdown restrictions meaning that the team was not able to, for instance, print the documents and work together with all stakeholders in a room—which may have been another way a sense of shared ownership or addressing power imbalances could have been achieved.

#### 3.1.3 Considerations of Communication

Having to engage only in physically distanced communication presented both benefits and challenges to the collaborative process. Perhaps the most salient benefit for participants related to the increased accessibility of being able to attend videoconferences remotely. One example from the data set in which a participant elaborated on this point is provided in Extract 2.

Extract 2.

People had harder conversations more effectively online than I've seen them having in‐person. And I wonder a little bit about whether there's something about the safety of me being in my house, and my safe space, that allowed that to happen. … Often we invite—and researchers are really bad at it—we invite people into space that we control to participate in a conversation. And that in itself is a power problem. Whereas … the other thing that happened in this forum was that all of those people who dress up for work … there were no suits and there were no ties and … we all knew that we actually secretly had our pyjama bottoms on or our trackies … so there was this sense of informality, or being in the space together and grappling in the space together.

Organisational inaccessibility has been recognised as an impediment to the facilitation of co‐production [16] and it seems that having to address accessibility through videoconferencing becoming a norm may have helped to break down these barriers (acknowledging of course it comes with other accessibility barriers including digital literacy, or access to functioning internet connections and devices). This may have created what has been conceptualised as a ‘third sphere’ [17]—that is, a place that is not controlled by any stakeholder, but that allows collaborators to come together to co‐produce free from the traditional constraints of their ‘spheres’ (i.e., the academic or health system sphere, or the advocacy or consumer sphere).

Accessibility benefits notwithstanding, the limiting of communication to socially distanced discussions also led to barriers in putting co‐production principles into practice. One of the facilitators recounted very practical difficulties communicating with two key groups.

Extract 3.

Although everyone was incredibly busy we still were able to get in contact with most key stakeholders. There were two groups, though, that we thought would be incredibly pivotal to the process that we couldn't contact—[a peak body representing older adults, and a health service provider for Aboriginal and Torres Strait Islander people]. The latter in particular I felt I was almost harassing them with multiple emails for weeks to the CEO and whatever email addresses I could find online. If it weren't during COVID I would have just driven there and figured out who to talk to. But that wasn't possible, and it was such a busy time for the health and social services sector caring for their own consumers, so I understand why this extra labour we were asking them to do got missed. Even though for me it seemed like *the* single most important task to do at the time, of course service providers had a lot more on their plate and were still worried about isolated consumers, people who had lost jobs, and uncertainty about safety of their staff.

The co‐planning and co‐design phases of the project continued with the other organisations who were able to be contacted. It seems this timing was critical because we also interviewed a member from the organisation who did engage in the collaboration during the third (i.e., co‐evaluation) stage. This person noted their manager “didn't respond because she nominated [the interviewee] as a contact person” but perhaps in the busy period of COVID their team had not forwarded them the email trails. Existing research has identified a lack of information exchange as an antecedent of miscoordination [18] and a simple solution, in this case, would have been a more formalised system to nominate one individual as a contact person to streamline information exchange. For some other groups involved, they said that it was ‘the first time [their group had] been involved in such a process, and [members] appreciated the communicative approach taken’. It is perhaps unsurprising that this subtheme about the role of communication in facilitating productive communication from the beginning of the project was related to stronger collaborations.

### 3.2 Principle 2: Power Differentials

The second principle of co‐production is concerned with addressing power relationships between all stakeholders [12]. Participants spoke about two aspects related to power: the permeability of power structures, and broader social and systemic imbalances.

#### 3.2.1 Permeability of Power Structures

One of the ways in which participants reported feeling a permeability in power related to the way in which discussions were held during the pandemic. As in Extract 2, above, videoconferences with people in their own houses created what felt for some like a neutral space. Previous research has found that consumers collaborating with health institutions can be left out of important discussions—and that in terms of physical space are often ‘not in the room’ with the people making decisions [19]. However, having senior people from the health service discuss over videoconference their concerns with consumers changed these power dynamics for some participants.

This is not to say, however, that other power dynamics were not present. One participant talked about a sense that there was ‘someone sitting at [the local health service] around this big table thinking about all of us in the community sector … and they're these great experts [and] our voice…had to be forced upon them’. Although a senior member of the local COVID‐19 response (who also held a role as an intensive care specialist) was present at all forum discussions to talk about issues from service and clinical perspectives, these data suggest that for some there remained an opacity in the whole project. The metaphor of a ‘big table’ removed from the community somewhat viscerally demonstrates how the power imbalance was felt by some. Others found this style of engagement to be breaking down barriers (such as in Extract 2 who noted that all being ‘in the space together’ in ‘pyjama bottoms’ equalised power relations even with this senior colleague).

Another finding related to the permeability of power structures related to the way that participants felt about facilitation being led by external academics not employed by the health service. From the perspective of the theoretical framework of co‐production, it seems that there could be both benefits and costs to independent people acting as facilitators for such projects. On one hand, this felt to some community partners that there was an increased distance between the service and their input on the project—and this may have exacerbated this sense of opacity. For instance, one participant noted: ‘I don't think there was any problem with [the academic lead] but I think you need to have presence of people … the doctors and nurses and decision makers [otherwise] they're not going to take it seriously’. On the other hand, independent facilitation meant that the project was coordinated by someone able to listen, who had a history of experience in collaboration, and a level of independence from the service (such that they did not have to push any particular agenda *other than* collaborating on the triage process). For instance, another participant noted the expertise of the academic lead was of benefit, stating that they were ‘an expert in doing this kind of collaboration and bringing it together’. Previous research has explored how aspiring allies may be able to use their power and privilege to make space for marginalised groups and individuals [20]; however, our findings contribute relevant conceptual insights into such roles given that even though the facilitator was independent of the health service, tensions still grew between the health services' goal (i.e., to learn how to allocate intensive care resources) and the concern of some collaborators that this would not be compatible with a human rights–based approach.

#### 3.2.2 Broader Social and Systemic Imbalances

While health systems globally were grappling with how to manage COVID‐19, particularly in the early months of the pandemic, there were concerns about how health services would triage and care for those at higher risk of severe disease. In particular, there were concerns about the exclusion of older adults and people with disabilities [21] and other groups with existing health inequities (such as Aboriginal and Torres Strait Islander communities in Australia). For some participants in the current study, they reported that members of their communities felt that the development of the triage process for intensive care was going to reproduce systemic imbalances against their communities. An example of this is shown in Extract 4, where a participant is talking about their impression the CEO of their community organisation had of the project:

Extract 4.

[to her] it was a token consultation to tick the First Nations box and we were never going to be taken into consideration … the triage process was always going to ignore Aboriginal people … I think she just thinks it was just another cursory consultation process which was with a preordained outcome and that to her that's just another … example of that stuff happening to Aboriginal interest around the table.

Extract 4 relates to an important consideration for health professionals wanting to collaborate with people from marginalised communities—some are likely to be understandably wary that any given project involving health professionals or services will not address power imbalances. The broader context of this extract was related to other advocacy work that this organisation's CEO does every day in the context of equity issues such as Aboriginal deaths in custody (and Aboriginal and Torres Strait Islander people and organisations in leadership roles are often overwhelmed by others seemingly wanting consultation). Thus, even when facilitators do not want just consultation, but want collaborators to take control of directly editing outputs (as in Extract 1), one barrier may be the way that they have been treated historically within other projects or contexts.

### 3.3 Principle 3: Development of Consumer Leadership

The final principle of co‐production with consumers relates to ensuring that consumer leadership and capacity are developed—and that co‐production ‘harnesses the knowledge and skills of everyone involved’ [12]. There were two themes developed through our analytic process in relation to this principle: the way that the triage process was transformed through this project, and valuing consumer expertise.

#### 3.3.1 Transformational Aspect of Co‐Production

Perhaps the most obvious outcome of consumer leadership over the development of the triage process is in the way that particular community partners completely transformed the direction and product of the triage process. An important outcome of the collaboration—particularly with partners from a range of marginalised groups, including the disability advocacy sector—was that human rights principles underpinned the approach taken in the triage process [8]. While many COVID‐19 triage processes from around the world do discriminate based on factors such as age [22], or underlying health conditions [23], the process developed for the ACT explicitly requires a non‐discriminatory approach. This includes making decisions not only based on immediate clinical conditions but also embeds other mechanisms to ensure a human rights basis. Such mechanisms include reasonable adjustments for patients (such as addressing accessibility needs or providing appropriate communication supports), requirements for unconscious bias training for clinicians and a review process for triage decisions. This required significant labour from community partners who had been telling facilitators this from the beginning of the process, as in Extract 5.

Extract 5.

One of the things that just really struck me as we were looking at drafts and as things are progressing was just how far there is still to go around culture around human rights, in particular. It was really interesting to see that the second to last draft was ‘human rights, non‐discrimination’ all the way through until there was a clause that was completely discriminatory.

As this participant states, even up to the penultimate draft of the triage process, a discriminatory clause about decisions based on age remained (even after having established that triage must be based on ensuring the protection of human rights). This was perhaps in part because of two major competing approaches to the development of the triage system: one (a resources‐based approach) from those working in intensive care who were worried about an extremely overwhelmed system and not having enough staff or ventilators to support all who might need them, and another (a human rights–based approach) from those particularly in the disability advocacy space who were worried that approaching the question with a focus on resources could perpetuate inequities. Factors supporting the eventual change of the outputs to remove the age‐based ‘tiebreaker’ included ensuring collaborators felt they were able to directly edit outputs (as per Extract 1), and the ongoing, iterative discussions. Again, without the transformation of the triage process led by the community partners, the product would have been completely different.

#### 3.3.2 Valuing Consumer Expertise

An important feature of co‐production between health service providers and service users is ensuring that everyone's contributions are valued—and the facilitators were informed by existing research about how healthcare consumers' experiential expertise is as valuable as health professionals' knowledge [8, 24], as seen, for instance, in the following extract from one of the facilitators.

Extract 6.

It's all good and well health practitioners telling patients to do something, or creating a service, or spending money on something that's no good if patients don't turn up, or it's not what they need, or they don't understand why we've done this and I think patients have an awful lot of insight. I've never had cancer, I've never had a baby, I'm not a male, I'm white, and … I think it's really important that we build things for all consumers … Consumers need power to say ‘actually, no, that's a really dumb idea’.

Valuing consumers' experience (and a diversity of experiences), as discussed in the Extract demonstrates the approach that facilitators took in this collaboration. Further, not just consulting with consumers, but ensuring consumers have ‘power to say’ how the process should unfold reflects the way consumer leadership in the development of the triage process was seen as critical.

## 4 Discussion

The current study emphasises that within a single project, the principles of co‐production can be met in some ways and not in others (as shown in Table 1 and throughout the qualitative results section). The results highlight ways that working towards the principles of co‐production created space for consumer, carer and community collaborators to transform the development of the COVID‐19 triage process from being centred on resources to being centred on human rights. From the perspective of the theoretical framework of co‐production [12], the findings illustrate ways in which the importance of each core principle (i.e., consumers are partners from the outset, power differentials are considered, and capacity is built) might be realised.

Academic literature about co‐production has been critiqued for being fragmented [25], and for being limited to aspirational or methodological concerns [13]. Much existing research has used the term co‐production to refer to individual‐level (i.e., an individual patient making decisions about their own health care or treatment) instances of participation in care [26]. The current study contributes to the literature by applying the theoretical framework of co‐production and exploring how its principles have been met (and not met) within a system‐level collaboration in developing a high‐stakes health policy.

The findings contribute to an understanding of co‐production by suggesting key factors to meet these core principles. Specifically, to ensure projects meet the principle of being partners from the outset, our findings highlight the importance of planning together and continuous collaboration (as in Extract 1), and of keeping channels of communication open (see Extracts 2 and 3). To meet the principle of addressing power relationships, our findings emphasise the importance of acknowledging broader social and systemic imbalances and considering ways to make power structures more permeable. The third principle is concerned with consumer leadership and capacity being developed through co‐production. In the context of the triage development, consumer leadership was developed through consumer expertise transforming the triage process (noting that in other projects or contexts lived experience expertise may in fact confirm existing foundations rather than transform them if they are already robust).

### 4.1 Implications

The findings of this study have implications that might inform the practices of those engaging in co‐produced projects. First, a key principle of co‐production is concerned with addressing power differentials. The current study contributes to the theoretical framework of co‐production given the nature of the evaluated project as a collaboration between people from several stakeholder groups and in a particular context where several key stakeholder groups were marginalised and systemically discriminated against in ways directly relevant to the project. Previous literature has referred to acts whereby health professionals use their power to create space for consumer leadership as ‘allyship’ [27]. The current study extends the conceptualisation of allyship by suggesting one way to engage in acts of allyship is to reduce power differences between consumers and other stakeholders within the health sector (who have traditionally held the power). Based on these findings, those who want to advocate for more robust consumer engagement in health systems are encouraged to consider how they might use their role to make power structures more permeable.

One example of how power imbalances during the development of the triage principles and process could have been made more permeable is that the co‐production phases could have been thought of as less linear. That is, during the co‐planning phase, collaborators agreed on the ‘problem’ to ‘solve’ being related to how to allocate intensive care resources if need exceeded capacity during the pandemic (see Table 1). As the project moved into subsequent phases, and as broader and deeper engagement was sought with consumer, carer and community groups, a fundamental concern arose (as in Extract 5) that a question focussed on resources might conflict with a human rights–based answer. Power imbalances could have been made more permeable by revisiting the co‐planning phase in a more iterative manner, providing opportunities to refine or adjust the ‘problem’ in a less linear way. Thus, another contribution of our findings is to encourage facilitators to consider how they navigate co‐production and to think of each phase in more iterative or cyclical ways to address power imbalances.

Second, the transformational aspects of the consumer‐led change to the triage process were significant. Through a lot of hard work, consumers and advocates ensured that the triage process was based on human rights principles and non‐discrimination—and this likely would not have happened without co‐production. Aligned with the principle of capacity building [12] when the facilitators positioned themselves as learners in co‐production activities, the expertise of consumers was transformative. In this project, this may have happened organically—or perhaps as a result of the way that COVID‐19 was novel and overwhelming at the time, that non‐consumer partners (who traditionally may have taken the lead) were more open to accepting that they needed to learn. Further, the final triage process developed was based on human rights—which could not have been achieved if consumer, carer and community groups' expertise was not truly valued. Indeed, it is hard to imagine a successful collaborative process without valuing this expertise. Practical ways in which this could be demonstrated in future initiatives include inviting collaborators to directly edit outputs (and ensuring they understand that it is a genuine invitation to do so with necessary practical or academic support as needed) or seeking ways to facilitate consumer leadership of collaborative processes.

Last, this project was high‐stakes, dealing with access to intensive care resources such as invasive ventilators, and as such there were some very difficult discussions for stakeholders about life and death (and in addition took place when a range of other COVID‐related stressors were high). The facilitators wanted to ensure that people at risk of severe disease or marginalised people who would be impacted by these decisions were part of the collaboration (as in Extract 6), but particularly marginalised groups were understandably wary because of the way their involvement has been made tokenistic in the past (as in Extract 4). The knowledge that marginalised people bring is often very different from the kinds of knowledge considered legitimate by institutions and systems [28]. Nonetheless, the approach taken in the development of the triage process in the ACT was that consumers' expertise was as important as health professionals' knowledge [8, 29]. We recommend health institutions demonstrate commitment to true co‐production and non‐tokenistic involvement so that all can benefit from consumers' experiential expertise.

Related to the above point, an important contribution this study makes to the broader literature about collaborative approaches relates to the groups and individuals identified as necessary for collaboration to be meaningful. Initiatives claiming to have done co‐production or other collaborative methods often make a conflation between collaboration being sought from particular groups because they are ‘marginalised’ and collaboration being sought from particular groups because they are *centrally impacted by this particular project*. In the context of COVID, for instance, Aboriginal and Torres Strait Islander people and older people are not just marginalised in this context but are disproportionately impacted by COVID and the way pandemic responses have been seen to uphold racist and ageist practices, and so their knowledge is crucially needed. One implication of this for future initiatives is to ensure that collaborations make space for and centre the experiences of people who are centrally impacted.

### 4.2 Future Directions

The study provided an opportunity to consider the role of external facilitation in the application of co‐production principles. The results of the current study suggest that some felt this created a greater distance and opacity between them and the service, while others pointed to the importance of expertise (that may not yet be present across health services) in engaging with the community. Certainly, the human rights focus of the outcome of the triage process was different from what health professionals working within the service would have expected. Whether or not independent facilitation helped to bring this about, previous research has suggested that in situations where consumers distrust systems, ‘credible intermediaries’ might redress this distrust [30]. Future research might consider more fully the implications of independent facilitators and the way that they might be able to challenge or may need to uphold certain power dynamics.

## 5 Conclusion

Our findings make an empirical and applied contribution to the literature about co‐production with healthcare consumers. Extending on the theoretical framework of co‐production, we describe stakeholders' perspectives about each core principle and provide recommendations for others who want to continue to strive to meet these principles. Although co‐production has been critiqued for becoming a buzzword or a merely aspirational idea, our findings demonstrate that initiatives can be assessed against how well they are meeting these core principles.

## Author Contributions

**Brett Scholz:** conceptualisation, data curation, formal analysis, writing–original draft, methodology, writing–review and editing, project administration. **Flick Grey:** conceptualisation, formal analysis, writing–review and editing. **Joyce Graham:** conceptualisation, formal analysis, writing–review and editing. **Imogen Mitchell:** conceptualisation, formal analysis, writing–review and editing. **Lucy Kirk:** conceptualisation, formal analysis, writing–review and editing. **Terri Warner:** conceptualisation, formal analysis, writing–review and editing.

## Ethics Statement

The study was approved by the ANU Human Research Ethics Committee (#2020/272).

## Consent

All participants consented to take part in the study.

## Conflicts of Interest

The authors declare no conflicts of interest.

## Data Availability

The data that support the findings of this study are available on request from the corresponding author. The data are not publicly available due to privacy or ethical restrictions.

## References

[1] V. Tangcharoensathien , M. T. Bassett , Q. Meng , and A. Mills , “Are Overwhelmed Health Systems an Inevitable Consequence of Covid‐19? Experiences from China, Thailand, and New York State,” BMJ 372 (2021): n83. 33483336 10.1136/bmj.n83 PMC8896039

[2] M. Z. Solomon , M. K. Wynia , and L. O. Gostin , “Covid‐19 Crisis Triage—Optimizing Health Outcomes and Disability Rights,” New England Journal of Medicine 383 (2020): e27. 32427434 10.1056/NEJMp2008300

[3] “Statement of Concern—COVID‐19: Human Rights, Disability and Ethical Decision‐Making, Disabled People's Organisations Australia,” 2020, https://dpoa.org.au/statement-of-concern-covid-19-human-rights-disability-and-ethical-decision-making/.

[4] K. Staley , J. Elliott , D. Stewart , and R. Wilson , “Who Should I Involve in My Research and Why? Patients, Carers or the Public?” Research Involvement and Engagement 7 (2021): 41. 34127074 10.1186/s40900-021-00282-1 PMC8202960

[5] K. Moodley , S. Rennie , F. Behets , et al., “Allocation of Scarce Resources in Africa During COVID‐19: Utility and Justice for the Bottom of the Pyramid?” Developing World Bioethics 21, no. 1 (2021): 36–43, 10.1111/dewb.12280. 32845575 PMC7461286

[6] J.‐J Zhang , X. Dong , G.‐H Liu , and Y.‐D Gao , “Risk and Protective Factors for COVID‐19 Morbidity, Severity, and Mortality,” Clinical Reviews in Allergy & Immunology 64 (2022): 90–107, 10.1007/s12016-022-08921-5. 35044620 PMC8767775

[7] R. Storen and N. Corrigan , “COVID‐19: A Chronology of State and Territory Government Announcements (Up Until 30 June 2020),” Parliament of Australia, Parliamentary Library, 2020, https://parlinfo.aph.gov.au/parlInfo/download/library/prspub/7614514/upload_binary/7614514.pdf.

[8] B. Scholz , L. Kirk , and I. A. Mitchell , Australian Capital Territory COVID‐19 Intensive Care Triage Principles and Process: Consumer, Carer, and Community Consultation Report (Canberra, Australia: Australian National University, 2020), http://hdl.handle.net/1885/227267.

[9] “COVID‐19 Pandemic Response,” ACT Government, July 9, 2020, https://www.hansard.act.gov.au/Hansard/9th-assembly/Committee-transcripts/covid17a.pdf.

[10] R. Dunston , A. Lee , D. Boud , P. Brodie , and M. Chiarella , “Co‐Production and Health System Reform—From Re‐Imagining to Re‐Making,” Australian Journal of Public Administration 68, no. 1 (2009): 39–52.

[11] B. Scholz , S. Stewart , A. Pamoso , S. Gordon , B. Happell , and B. Utomo , “The Importance of Going Beyond Consumer or Patient Involvement to Lived Experience Leadership,” International Journal of Mental Health Nursing 33, no. 1 (2024): 1–4, 10.1111/inm.13282. 38131453

[12] C. Roper , F. Grey , and E. Cadogan , “Co‐Production: Putting Principles Into Practice in Mental Health Contexts,” Melbourne School of Health Science, 2018, https://healthsciences.unimelb.edu.au/__data/assets/pdf_file/0007/3392215/Coproduction_putting-principles-into-practice.pdf.

[13] E. Turnhout , T. Metze , C. Wyborn , N. Klenk , and E. Louder , “The Politics of Co‐Production: Participation, Power, and Transformation,” Current Opinion in Environmental Sustainability 42 (February 2020): 15–21.

[14] R. A. Stebbins , Exploratory Research in the Social Sciences (London, UK: Sage, 2001).

[15] V. Braun and V. Clarke , “Using Thematic Analysis in Psychology,” Qualitative Research in Psychology 3, no. 2 (2006): 77–101.

[16] M. Sicilia , A. Sancino , T. Nabatchi , and E. Guarini , “Facilitating Co‐Production in Public Services: Management Implications From a Systematic Literature Review,” Public Money & Management 39, no. 4 (2019): 233–240.

[17] C. Dierckx , L. Hendricks , S. Coemans , and K. Hannes , “The Third Sphere: Reconceptualising Allyship in Community‐Based Participatory Research Praxis,” Qualitative Research in Psychology 18, no. 4 (2021): 473–497, 10.1080/14780887.2020.1854402.

[18] J. A. Aunger , R. Millar , J. Greenhalgh , R. Mannion , A.‐M. Rafferty , and H. McLeod , “Why Do Some Inter‐Organisational Collaborations in Healthcare Work When Others Do Not? A Realist Review,” Systematic Reviews 10 (2021): 82. 33752755 10.1186/s13643-021-01630-8 PMC7984506

[19] B. Scholz , J. Bocking , P. Hedt , V. N. Lu , and B. Happell , “‘Not in the Room, but the Doctors Were’: An Australian Story—Completion Study About Consumer Representation,” Health Promotion International 35, no. 4 (2020): 752–761, 10.1093/heapro/daz070. 31325360

[20] P. Juntanamalaga , B. Scholz , C. Roper , and B. Happell , “‘They Can't Empower Us’: The Role of Allies in the Consumer Movement,” International Journal of Mental Health Nursing 28, no. 4 (2019): 857–866, 10.1111/inm.12585. 30834660

[21] B. Chen and D. M. McNamara , “Disability Discrimination, Medical Rationing and COVID‐19,” Asian Bioethics Review 12 (2020): 511–518. 32901207 10.1007/s41649-020-00147-x PMC7471485

[22] J. Rueda , “Ageism in the COVID‐19 Pandemic: Age‐Based Discrimination in Triage Decisions and Beyond,” History and Philosophy of the Life Sciences 43, no. 3 (2021): 91. 34258692 10.1007/s40656-021-00441-3 PMC8276843

[23] H. Michielsen , I. De laet , J. Van Bastelaere , J. Huygh , K. Bervoets , and N. Van Regenmortel , “A Retrospective Evaluation of Three Ethical Triage Tools for the Allocation of ICU Resources During the First Wave of the COVID‐19 Pandemic,” Journal of Critical Care 67 (2022): 200–206, 10.1016/j.jcrc.2021.09.022. 34642069 PMC8501390

[24] B. Happell , S. Gordon , J. Bocking , et al., “How Did I Not See That? Perspectives of Non‐Consumer Mental Health Researchers on the Benefits of Collaborative Research With Consumers,” International Journal of Mental Health Nursing 27, no. 4 (2018): 1230–1239, 10.1111/inm.12453. 29527786

[25] F. Fusco , M. Marsilio , and C. Guglielmetti , “Co‐Production in Health Policy and Management: A Comprehensive Bibliometric Review,” BMC Health Services Research 20 (2020): 504. 32503522 10.1186/s12913-020-05241-2 PMC7275357

[26] R. Palumbo , “Contextualizing Co‐Production of Health Care: A Systematic Literature Review,” The International Journal of Public Sector Management 29, no. 1 (2016): 72–90.

[27] B. Happell and B. Scholz , “Doing What We Can, but Knowing Our Place: Being an Ally to Promote Consumer Leadership in Mental Health,” International Journal of Mental Health Nursing 27, no. 1 (2018): 440–447, 10.1111/inm.12404. 29171920

[28] T. Brandsen , “Vulnerable Citizens: Will Co‐Production Make a Difference,” in The Palgrave Handbook of Co‐Production of Public Services and Outcomes, eds. E. Loeffler and T. Bovaird (London, UK: Palgrave Macmillan, 2020), 527–539.

[29] B. Scholz , J. Bocking , and B. Happell , “Improving Exchange With Consumers Within Mental Health Organizations: Recognizing Mental Ill Health Experience as a ‘Sneaky, Special Degree’,” International Journal of Mental Health Nursing 27, no. 1 (2018): 227–235, 10.1111/inm.12312. 28145617

[30] H. Li , “Communication for Coproduction: Increasing Information Credibility to Fight the Coronavirus,” The American Review of Public Administration 50, no. 6–7 (2020): 692–697.

